# Is it Safe to Perform Laparoscopic Cholecystectomy for Acute Calculus Cholecystitis within 7 Days Following Symptom Onset?

**DOI:** 10.31729/jnma.3655

**Published:** 2018-12-31

**Authors:** Rajesh Poudel

**Affiliations:** 1 Department of Surgery, Universal College of Medical Sciences, Bhairahawa, Nepal

**Keywords:** *acute*, *cholecystectomy*, *cholecystitis*, *early*, *safe*

## Abstract

**Introduction:**

Although operation within “golden 72 hours” from the onset of symptoms has been suggested for acute calculus cholecystitis, such early surgery is hardly possible in clinical practice because of variable timing of presentation. The aim of this study is to compare the outcomes of patients undergoing laparoscopic cholecystectomy within 72 hours of symptom onset with patients undergoing surgery after 72 hours up to 7 days of symptom onset for acute calculus cholecystitis.

**Methods:**

This is a descriptive cross-sectional study carried out from November 2016 to July 2018. Patients with acute calculus cholecystitis were divided in two groups according to the onset of symptoms. Main outcomes measured were conversion rate, duration of surgery, length of hospital stay and intraoperative complications.

**Results:**

Total 64 patients were evaluated. Among which 18 (28.1%) underwent surgery within 72 hours of onset of symptom. Around 46 (71.9%) underwent surgery after 72 hours of symptom onset. On bivariate analysis there were no significant differences in mean duration of surgery, hospital stay and conversion to open surgery between two groups.

**Conclusions:**

Early laparoscopic cholecystectomy is a safe procedure when done within 7 days of symptom onset. There were no significant difference in conversion rate, operative time, hospital stay, morbidity and mortality.

## INTRODUCTION

The appropriate timing for laparoscopic cholecystectomy in the treatment of acute cholecystitis remains controversial. Early open cholecystectomy had previously been established as the preferred management of acute cholecystitis.^1^ With the advent of laparoscopic surgery, the benefits of early surgery have been the subject of some contention.^2^

Studies have indicated early laparoscopic surgery to be a safe option in acute cholecystitis.^3–8^ Laparoscopic cholecystectomy is considered to be the standard of care if the patient is seen within 72 hours of the attack.^6^ Although operation within the “golden 72 hours” from the onset of symptoms has been suggested, such early surgery is hardly possible in clinical practice because of variable timing of presentation.

The aim of this study is to compare the outcomes of patients undergoing laparoscopic cholecystectomy within 72 hours of symptom onset with patients undergoing surgery after 72 hours up to 7 days of symptom onset for acute calculus cholecystitis.

## METHODS

This is descriptive cross-sectional study carried out in Universal College of Medical Sciences, Bhairahawa, Nepal from November 2016 to July 2018. The ethical approval was taken from Institutional Review Board of Universal College of Medical Sciences, Bhairahawa. The diagnosis and assessment of acute cholecystitis were performed in accordance with the diagnostic criteria of the Revised Tokyo Guidelines published in 2013.^9^ All patients diagnosed with acute calculus cholecystitis were included in the study. Those patients who have symptoms >7 days, Pregnancy or Malignancy were excluded from the study.

After fulfilling the inclusion and exclusion criteria, patients were given adequate explanation about the study and assured of full confidentiality. Patients were divided in two groups according to the onset of symptoms. Patient with onset of symptoms less than 72 hours were kept in one group (Group A) and those patients whose symptom of onset is more than 72 hours but less than 7 days were kept in other (Group B). Patients' demographics, clinical characteristics, preoperative data, surgical intervention, intraoperative findings, biliary injury, intraoperative bleeding, conversion to open surgery, duration of operation, length of hospital stay, outpatient follow up, surgical complications and mortality were recorded. Outcomes measured were conversion rate, duration of surgery, surgical site infection, length of hospital stay. Other complications measured were bile duct injury, intractable bleeding, post-operative fever and mortality. The data were entered using SPSS software. Categorical variable were compared with Chi-Square or Fisher exact test and continuous variables were compared with independent samples T-test and Mann-Whitney U test, where appropriate. 95% confidence interval was taken and P value less than 0.05 was considered statistically significant.

## RESULTS

Total of 64 patients were evaluated, among which 18 (28.1%) underwent surgery within 72 hours of onset of symptom. About 46 (71.9%) underwent surgery after 72 hours of symptom onset. Baseline characteristics of patients in two groups were compared. There were no significant differences in between these two groups in regard to mean age, sex, American Society of Anaesthesiologists' (ASA) grade and White Blood Cell (WBC) count preoperatively (Table 1).

**Table 1:** Baseline characteristics of patients in two groups.

Variable	Group A (<72 hours)	Group B (>72 hours)	P
Age	42.78±16.79	46.96±16.03	0.372
Female Sex	14	36	0.967
ASA (>II)	6	11	0.449
WBC	11405.56±1051.31	12363.04±13662.7	0.639
ASA: American Society of Anaesthesiologists', WBC: white blood cell.			

On bivariate analysis, there were no significant differences in mean duration of surgery between patients in Group A and Group B (81.56±42.92 minutes vs. 83.29±36.85, P = 0.88, 95% confidence interval (CI), -24.53 to 21.075). There were no significant differences between hospital stay in two groups (3.45 vs 3.85 days, P = 0.58, 95% CI, -1.778 to 0.990). Conversion to open surgery was high in patients undergoing surgery after 72 hours (21.73% vs 5.55%) but this difference was not statistically significant P = 0.092. Surgical site infection (SSI) was higher in group A but this was not of statistical significance (P = 0.338) (Table 2).

**Table 2:** Outcomes in two groups.

Variables	Group A (<72 hours)	Group B (>72 hours)	P
Duration of surgery (minutes)	81.56±42.92	83.29±36.85	0.88
Conversion	1 (5.55%)	10 (21.73%)	0.092
SSI	3 (16.66%)	3 (6.52%)	0.338
Length of hospital stay (days)	3.45±2.08	3.85±1.95	<0.58

There was one case of intraoperative bile duct injury and one intractable bleeding in group B and group A respectively. Two patients developed fever due to chest infection in group A. There was no mortality in any of the group (Table 3).

**Table 3:** Post-Operative Complications.

Variable	Group A (<72 hours)	Group B (>72 hours)
Intraoperative bile duct injury	0	1
Intractable bleeding	1	0
Post-op fever	2	0
Mortality	0	0

## DISCUSSION

Laparoscopic cholecystectomy is the gold standard in the therapy of symptomatic cholelithiasis and has gained broad acceptance.^10^ During the early years, acute cholecystitis was a contraindication for laparoscopic treatment because of inflammation and oedema, which made laparoscopic cholecystectomy unsafe.^11^

Randomized trials performed by Lo CM et al. and Lai PB et al. in late 1990s comparing early versus delayed laparoscopic cholecystectomy for management of acute cholecystitis showed that the urgent procedure is safe compared with delayed surgery.^7,12^ Since then multiple trials have been done and concluded that early laparoscopic cholecystectomy can be performed safely for acute cholecystitis.

A Cochrane review of five randomised trials for acute cholecystitis showed that early laparoscopic cholecystectomy was as safe as delayed laparoscopic cholecystectomy with a shorter hospital stay. No mortality was reported in any of the trials. The definition of early surgery varied between 4 and 7 days of onset of symptoms.^13^ Despite the acceptance of early cholecystectomy as a safe procedure for acute cholecystitis, definition of early is not well defined. In our study, most of the patients with acute calculus cholecystitis presented after 72 hours of symptoms (46 vs 18). Our study showed no significant differences in regard to conversion to open surgery, length of hospital stay, surgical site infection and duration of surgery in these two groups. Conversion to open surgery in patients who had undergone surgery after 72 hours seems higher when compared with the patients whose surgery were done within 72 hours of symptom onset (21.73% vs 5.55%). This difference was not statistically significant. One patient who had bile duct injury in our study was converted to open and found to have Mirrizi syndrome type 3 that was missed preoperatively. There was no mortality in any of the group.

In a study done by Hamad Hadi Al-Qahtani et al. in Saudi Arabia where they compared patient who had undergone early laparoscopic cholecystectomy within 7 days of symptom onset to delayed cholecystectomy group (Surgery after 6 weeks), they found that early Laparoscopic cholecystectomy within 7 days of symptom is a safe procedure. It resulted in the reduction of the length of hospital stay without significant increase in conversion rate or bile duct injuries.^14^ Another similar study done by Baiju Senadhipan et al. in Kerala, India where they compared 30 patients who underwent laparoscopic cholecystectomy within 48 hour of onset of symptoms (group 1) to 60 patients who underwent surgery between 48 hours and 6 weeks of onset of symptoms (group 2). They concluded that laparoscopic cholecystectomy can safely be performed at any time after the onset of acute cholecystitis.^15^

Another study done by Isamil et al. in Turkey, where they reviewed Medical charts of 172 patients who underwent early cholecystectomy after 72 hour and within 7 days of index admission with a diagnosis of acute cholecystitis between August 2009 and April 2014 where they concluded that early cholecystectomy performed by experienced surgeons after 72 hours of admission and within 7 days may be feasible and safe procedure.^16^ Although operation within the “golden 72 hours” from the onset of symptoms has been suggested, such early surgery is hardly possible. Presentation might be delayed because of various factors. We found that early laparoscopic cholecystectomy can safely be performed after 72 hours of symptom onset within seven days when compared to the patients who had undergone surgery within 72 hours of symptom onset.

There are several limitations to this study. Since this is single institutional study and all surgeries were performed by single surgeon, outcome might not be generalised. Long term follow up was not done, so incidence of late post-operative biliary stricture were not recorded.

## CONCLUSIONS

Early laparoscopic cholecystectomy is a safe procedure when done within 7 days of symptom onset. There is no significant difference in conversion rate, operative time, hospital stay, morbidity and mortality.
